# A New Concept for Modeling Phase Transformations in Ti6Al4V Alloy Manufactured by Directed Energy Deposition

**DOI:** 10.3390/ma14112985

**Published:** 2021-05-31

**Authors:** Jérôme Tchoufang Tchuindjang, Hakan Paydas, Hoang-Son Tran, Raoul Carrus, Laurent Duchêne, Anne Mertens, Anne-Marie Habraken

**Affiliations:** 1 Aerospace and Mechanical Engineering, Metallic Materials Science, University of Liège, Quartier Polytech 1, Allée de la Découverte 9 (B52), B-4000 Liège, Belgium; hpaydas@uliege.be (H.P.); anne.mertens@uliege.be (A.M.); 2 Urban and Environmental Engineering, Materials and Solid Mechanics, University of Liège, Quartier Polytech 1, Allée de la Découverte 9 (B52), B-4000 Liège, Belgium; hstran@uliege.be (H.-S.T.); l.duchene@uliege.be (L.D.); anne.habraken@uliege.be (A.-M.H.); 3 Sirris Research Centre (Liège), Rue Bois Saint-Jean 12, B-4102 Seraing, Belgium; Raoul.Carrus@Sirris.be; 4 Fund for Scientific Research (F.R.S-FNRS), rue d’Egmont 5, B-1000 Bruxelles, Belgium

**Keywords:** directed energy deposition, microscopy and microanalysis techniques, Ti6Al4V alloy, phase transformation mechanisms, thermal modeling, experimental validation

## Abstract

The microstructure directly influences the subsequent mechanical properties of materials. In the manufactured parts, the elaboration processes set the microstructure features such as phase types or the characteristics of defects and grains. In this light, this article aims to understand the evolution of the microstructure during the directed energy deposition (DED) manufacturing process of Ti6Al4V alloy. It sets out a new concept of time-phase transformation-block (TTB). This innovative segmentation of the temperature history in different blocks allows us to correlate the thermal histories computed by a 3D finite element (FE) thermal model and the final microstructure of a multilayered Ti6Al4V alloy obtained from the DED process. As a first step, a review of the state of the art on mechanisms that trigger solid-phase transformations of Ti6Al4V alloy is carried out. This shows the inadequacy of the current kinetic models to predict microstructure evolution during DED as multiple values are reported for transformation start temperatures. Secondly, a 3D finite element (FE) thermal simulation is developed and its results are validated against a Ti6Al4V part representative of repair technique using a DED process. The building strategy promotes the heat accumulation and the part exhibits heterogeneity of hardness and of the nature and the number of phases. Within the generated thermal field history, three points of interest (POI) representative of different microstructures are selected. An in-depth analysis of the thermal curves enables distinguishing solid-phase transformations according to their diffusive or displacive mechanisms. Coupled with the state of the art, this analysis highlights both the variable character of the critical points of transformations, and the different phase transformation mechanisms activated depending on the temperature value and on the heating or cooling rate. The validation of this approach is achieved by means of a thorough qualitative description of the evolution of the microstructure at each of the POI during DED process. The new TTB concept is thus shown to provide a flowchart basis to predict the final microstructure based on FE temperature fields.

## 1. Introduction

Understanding the mechanisms that trigger phase transformations during additive manufacturing (AM) remains a major issue, as the final microstructure strongly influences the mechanical properties. Numerous studies have investigated the influence of processing parameters on the melt pool temperature and geometry [1,2,3,4,5,6], the solidification modes [1,2,6,7,8,9,10], the grain size and texture [3,5,7,8,9,11,12,13], as well as internal defects, distortions and residual stresses [1,6,10,11,12,14,15]. However, these approaches centered on macroscopic properties of AM parts neglect the understanding of the microstructure genesis during manufacturing. Attempts have also been made to optimize the microstructure of as-built AM parts, either via in situ tailoring [12,16,17], through post heat treatments [3,18], by changing the chemical composition [7,19], or by combining rolling deformation with the chosen AM process [20]. However, the improvements of the final properties remain questionable [12,21,22,23]. In fact, each AM process produces different phases in type, morphology and volume fraction [13,18,22,24,25]. Those microstructures lead to distinct mechanical properties that can be potentially improved by post heat treatments [10,16,18,24,26]. Nevertheless, none of these approaches focus on the accurate prediction and control of microstructural evolution during AM.

Analytical approaches and finite element (FE) simulations, on the other hand, can predict macroscopic features, internal flaws, residual stresses and microstructures [27,28,29,30]. FE macroscopic models used on their own or associated with the cellular automaton (CA) model can predict the thermal history, the melt pool geometry and the grain size and morphology during AM. CA is, for instance, used for 3D β-grain solidification simulation [31,32,33]. However, for subsequent solid-phase transformations, this approach remains irrelevant. Existing kinetic models consider fixed transition points and ignore the effect of thermal gradient strongly influencing both the mechanism and the kinetics of transformations (see literature review Section 2). Several works based on the partial exploitation of simulated thermal histories during the solidification or the final cooling stages can be found, but almost none of them are dealing with the evolution of the microstructure during AM [11,14,27,34].

In Ti6Al4V alloy, the process parameters, the temperature and the microstructure evolution have already been correlated for welding and laser hardening processes [15,29,30,35,36,37]. However, these cases are characterized by single cycle and high incident energies (IE) leading to low thermal gradients, while AM processes face complex cycles with lower IE and higher thermal gradients [28,38,39,40]. A macroscopic model to determine the influence of processing parameters on both density and mechanical properties was established for instance by [41]; however, it is not suitable for the prediction of microstructure evolution during AM.

The first trials of “replicated” thermal histories of AM were obtained by resistive heating and air cooling, thus leading to relatively slow heating and cooling rates [9,42,43], which is still far away from the steep thermal gradients achieved in AM [7,35,39,44]. In addition, there is currently no consensus on the values of the critical cooling rates, or on the transition points for the displacive martensite transformation and the diffusion-like β→α transformation upon cooling [4,11,18,44,45,46,47,48]. Similarly, a fixed value of *β_transus_* is often used for the completion of reverse transformation during heating, regardless of the heating rate $\dot{T}$ [15,29,34,43,45,48,49,50]. The recent work by Liu and Shin [33] combines multi-physics models to predict grain size and orientation, and phase distributions in single-layer DED deposits. In this study, macro and microstructures in the fusion zone and heat-affected zone (HAZ) are compared, but not the evolution of the microstructure. The phase amount is validated based on the rules of mixtures yielding the overall hardness. However, hardness prediction seems of poor accuracy, as shown in Section 2.4. To the author’s knowledge, there is currently no model taking into account the effect of $\dot{T}$ on either the mechanism or the kinetics of reverse transformations $\alpha /\alpha^{\prime }\to \beta$. In many studies, martensitic transformation is simply ignored during simulations [27,28,37,39], and when the final microstructure appears complex, an accurate validation based on precise microstructural characterization is often lacking [28,33,34,43,45]. The work from Xu et al. [16] is an attempt to experimentally describe structure evolution under the Laser powder bed fusion (L-PBF) process, which leads to a final martensitic microstructure. The authors suggest a sequence for martensite formation, highlighting the presence of twins that may influence both the nucleation and growth, but also the size and the distribution of martensite laths. The key parameter that is assumed to trigger the phase transformation is the peak temperature within the solid state. Nevertheless, this approach is based on projected thermal histories which are not simulated. Moreover, the microstructure is described in the final as-built conditions, without validation of its actual evolution during AM process.

Experimental studies and numerical simulations of AM processes too often remain separated, preventing a correct understanding of the microstructural evolution during manufacturing [40]. Relying on extensive experimental work and a careful literature review on the mechanisms that trigger solid-phase transformations in Ti6Al4V alloy, this study defines a clear flowchart and quantitative threshold values to improve phase predictions by FE simulations. This approach adapts continuous heating transformations (CHT), continuous cooling transformations (CCT) and time–temperature transformations (TTT) diagrams to better account for the ultra-fast heating and cooling rates and the local variations of thermal histories typical of AM processes.

The state-of-the-art is reviewed in Section 2. Section 3 describes the experiments and reminds the used FE thermal model [5,38]. Section 4 is devoted to new results, i.e., simulated thermal curves within three points of interest (POI). The main novelty of this paper is introduced in Section 5, where the new tool for microstructure prediction, the time-phase transformation-block (TTB) concept is described and applied on the three POIs of the experiment sample. A graphical synthesis allowing any interested reader to exploit the concept of TTB is provided at the end of Section 5 prior to drawing the main conclusions (Section 6).

## 2. Solid Phase Transformation Mechanisms and Kinetics—State of the Art

Hereafter, the specific metallurgical phenomena associated with the steep thermal gradients experienced during AM are highlighted. The focus is on the phase transformation mechanisms in the solid state more than on the solidification and remelting sequences. A distinction is made between non-isothermal and isothermal transformations. For the former, both heating and cooling modes are considered, while for the isothermal transformations, the focus is on the holding temperature and duration.

In addition, the hardness values reported on Ti6Al4V in different metallurgical states are reestablished, to highlight the limits in the interpretation of this parameter for characterizing the microstructure. Moreover, current kinetic models are reviewed, showing that to apply them as such to AM is inappropriate.

### 2.1. Continuous Heating Transformations

If the starting phase is α, the beginning and end points of the reverse transformation into β, referred to as α_transus_ and β_transus_, will increase with $\dot{T}$ [30,37,42,51]. For instance, shifts of 169 and 190 °C above the “equilibrium β_transus_” have been found for $\dot{T}$ of 43 and 100 °C/s, respectively, to achieve complete transformation into β upon heating [23,37]. Diffusion of V from α into β is reported to control the kinetics of this phase transformation, inducing changes in the lattice parameters of the two phases [16,23,24,35,51]. In addition, any β retained at a low temperature will remain untransformed upon heating.

**Figure 1:**
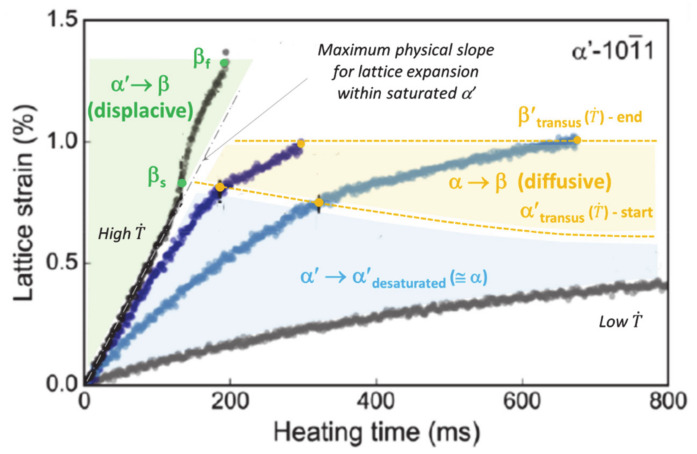
Influence of heating rate ($\dot{T}$) on the mechanism and transition points of the reverse transformations of α′, as given by the lattice parameter evolution (adapted with permission from ref. [52]. Copyright 2017 Springer Nature).

If the starting phase is α′, a steep increase in the temperature leads to a reverse displacive transformation (RDsT) α′ → β before the remelting of β above the liquidus [2,52]. No diffusion occurs, and the parent phase remains supersaturated up to complete transformation into β. This statement fits with the observation by Kenel et al. [52] that ultra-high heating rates result in the highest rate of expansion of the lattice parameter in the α′ phase (left-hand side of Figure 1). However, at lower heating rates, a high mobility of substitutional alloying elements promotes reverse diffusive transformation (RDfT) in Ti6Al4V [23]. It is thus assumed that α′ → β reverse transformation is displacive (RDsT) above a critical heating rate (CHR) $\dot{T}$ of 20 °C/s and diffusive (RDfT) below this CHR. For RDfT, the following sequence is achieved: α′ → α (+β) → β. Note that the beginning and the end of the RDsT, called β_s_ [53] and β_f_ (Figure 1), are distinct from β_transus_. The driving force for the RDfT of α′ is controlled by V diffusion [3,16,24], which also depends on $\dot{T}$. Both the onset and the endset of diffusional α′/α → β transformation, referred to as α′_transus_ and β′_transus_, respectively, will increase with $\dot{T}$. Note that β′_transus_ is higher than the equilibrium β_transus_. The decrease in the expansion rate of the α′ lattice at lower heating rates (bottom of Figure 1) is assumed to correspond to the desaturation of the martensite lattice [3,52].

### 2.2. Continuous Cooling Transformations

Phase transformations under continuous cooling conditions are usually represented by CCT diagrams that depend on the specific alloy and the applied solution treatment. In this regard, there are very few CCT diagrams for Ti6Al4V [1,26,29,43,47,54], and most of them are linked to one among the following two examples.

**Figure 2:**
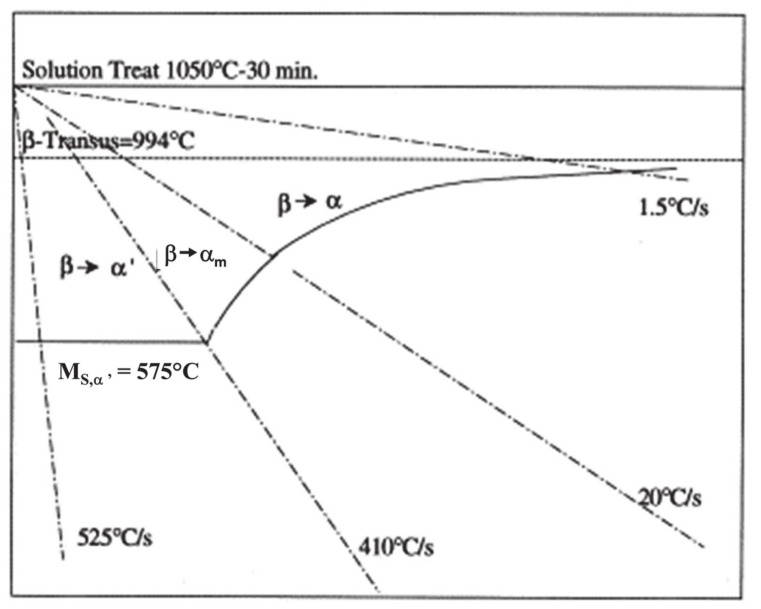
Schematic CCT diagram with only the starting points of the transformations of parent β phase and their related critical cooling rates $\left|\dot{T}\right|$. The apparent variation of starting temperatures for both α_m_. and α phases with $\left|\dot{T}\right|$, and the non-dependence of this same point for α′ with $\left|\dot{T}\right|$, suggest a DDfT and DDsT, respectively, for the related transformation products. Adapted with permission from ref. [54]. Copyright 1998 Elsevier.

The CCT diagram (Figure 2) that was established in 1998 corresponds to a simple sketch for which extended experimental validations are still missing [54]. The fields for the various phases are incompletely defined: only the starting points of the phase transformations are illustrated, while the end points are not shown. Moreover, the clear separation between the fields related to α_m_ and α′ suggests that these two phases may be distinctly formed [43]. The M_s,α′_ temperature associated with the martensitic transformation of the β phase seems too low, according to later publications, as reported hereafter.

**Figure 3:**
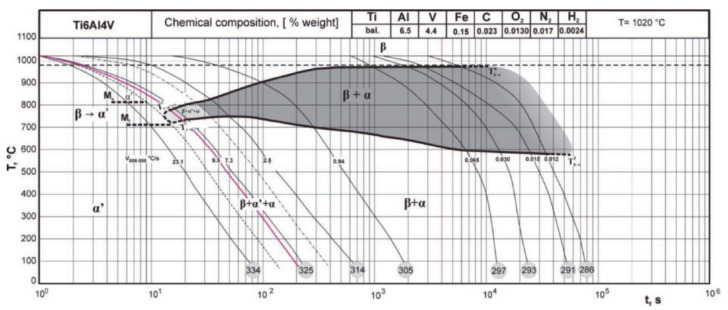
Experimental CCT diagram for Ti6Al4V, with both M_s,α′_ and M_f,α′_ values. Reprinted with permission from ref. [47]. Copyright 2011 PAN Journals PAS.

Thanks to an extended experimental validation, in 2011, the CCT diagram in Figure 3 [47] presents both the starting and end points of the transformation for the different phases. The values related to martensitic transformation appear relevant as explained hereafter and the location of phase fields is consistent with the transformation mechanisms. When $\left|\dot{T}\right|$ increases, the β_transus_ corresponding to the start of diffusive transformation decreases and the transformation range becomes narrower. No mention is made of α_m_.

When the starting phase is β, either direct diffusion transformation (DDfT) or direct displacive transformation (DDsT) occurs according to $\dot{T}$ [1,26,43,47,54,55]. For $\dot{\left|T\right|}$ lower than 20 °C/sec, DDfT yields an α/β structure, with a basket-weave (α/β_W_) or a colony morphology (α/β_C_), for higher or lower $\dot{\left|T\right|}$, respectively [3,18,47,54]. The starting point of the DDfT is given by β_transus_, which decreases from its equilibrium value with increasing $\dot{\left|T\right|}$ [42]. When the end point of the DDfT is reached, the transformation stops and a retained fraction of β remains (β_ret_) [16,30,33,45]. For $\left|\dot{T}\right|$ higher than 20 °C/s, the mechanism changes to DDsT generating α_m_ at grain boundaries, and/or α′ inside β grains. The coexistence of αm and α′ occurs for 20 < $\;\left|\dot{T}\right|$ < 410 °C/s [33,40,43,44,56], whereas only α′ is present for $\left|\dot{T}\right|$ > 410 °C/s [2,21,29,33,43,44]. The start point for α_m_ formation (called M_s,αm_) is 893 °C [17]. No mention exists for the end point of α_m_ formation. Clearly, no consensus exists for the starting point M_s,α′_ and the end point M_f,α′_ of the β → α′ transformation. Their values are shown hereafter:

- M_s,α__’_, 575 °C [22,29,39,54], 650 °C [7,15,35,36,48,52], 780 to 851 °C [1,19,38,47,57] or 915 °C [11,56,58];
- M_f,α__’_, 800 °C [59], 710 °C [47], 690 °C [58], 650 °C [1,19] or 400 °C [48]; M_f,α__’_ is also often assumed to be close to or lower than room temperature.

According to [60], the temperature gap between M_s,α__’_ and M_f,α__’_ should be small. Some amount of untransformed β_ret_ (with strongly distorted grains) remains after rapid cooling, due to high solute concentration and large undercooling [19,26,30,60,61,62].

### 2.3. Isothermal Transformations

**Figure 4:**
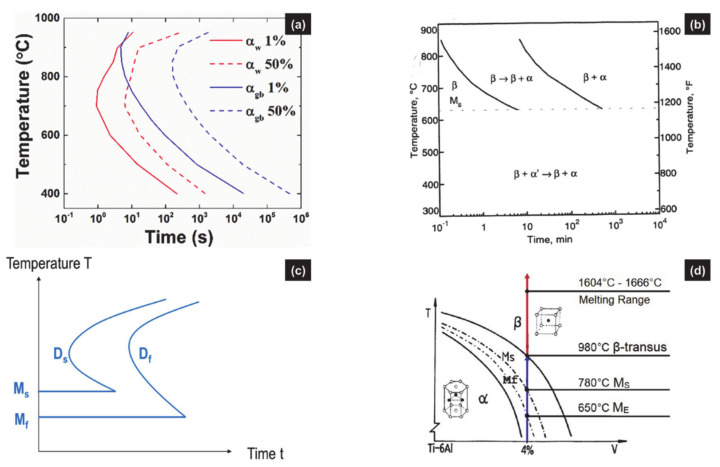
Examples of TTT diagrams for Ti6Al4V; (**a**) simulated TTT diagrams for α/β_W_ and α_GB_ (respectively annotated α_W_ and α_GB_) resulting from the diffusional phase transformation of β. (reprinted from ref. [56]); (**b**) TTT diagram of Ti6Al4V after solution annealed at 1020 °C and direct quenching to reaction temperatures showing DDfT and DDsT and M_s,α__’_ at 625 °C (reprinted from ref. [63]); (**c**) sketch of a TTT diagram plotting both a diffusional transformation (D) and a martensitic transformation (M), with their related starting curves D_s_ and M_s_, and the finishing curves D_f_ and M_f_ (reprinted from ref. [56]); (**d**) schematic pseudo-binary diagram of Ti6Al4V showing equilibrium metastable phase fields, with their related crystal structure and transition points (reprinted with permission from ref. [19]. Copyright 2019 Elsevier).

Isothermal transformation after cooling assumes a starting temperature higher than the soaking temperature and is usually linked to the parent phase β in AM of Ti6Al4V (Figure 4). Isothermal transformation after heating stage assumes heating up to a temperature where the sample is maintained and should be related to either α or α′ as starting phases, the latter being more relevant in AM.

Usually, isothermal transformations involve an incubation time before the start of any reaction. The different stages related to both activation energies and diffusion of species are illustrated in TTT diagrams (Figure 4a). Then, starting with β, the transformation products correspond to α_GB_ or α/β_W_, both resulting from a DDfT. A DDsT yielding α_m_ or α′ should, however, be expected as the quenching temperature falls into the thermal range for martensitic transformation (Figure 4b). Such a case is very often ignored [18,42,44,45], as confirmed by both simulated and experimental TTT diagrams [11,15,35,44]. To the authors’ knowledge, the only TTT diagram which combines DDfT and DDsT is a sketch where no value is defined for the beginning nor the end of the transformations (see Figure 4c) [56]. Otherwise, one can find the pseudo-binary diagram, where equilibrium phases coexist with unexpected metastable phases (Figure 4d).

With α′ martensite as the starting phase, a decomposition that is ascribed to a diffusion controlled phenomenon occurs, which has mainly been investigated experimentally [3,16,22,24]. The only existing model for the decomposition of martensite is the one established by Mur et al. [59]. However, this model has several limitations discussed in Section 2.5.

The case relating to a mixed α + β as starting phases will not be considered here, because it is rather specific to thermomechanical treatments carried out on the conventional alloy.

### 2.4. Microstructures and Hardness in Ti6Al4V

To see how considering only hardness measurement can be misleading to identify the phase distribution within a Ti6Al4V microstructure, a literature review is presented in Table 1 and Table 2.

A bimodal structure seems to exhibit a lower hardness. A small increase in hardness can be achieved under stress-relief treatment when starting from martensite, provided the tempering temperature is not too high (Table 1). Conversely, a decrease in both the strength and hardness is often expected from subsequent heat treatments carried out on martensite under high temperature.

Nevertheless, other features can also influence the hardness of Ti6Al4V processed by AM, such as the amount of interstitial elements (O, C and N) within the raw material, or contamination that is due either to inadequate storage conditions, to the reuse of powders, or to poor atmospheric protection conditions during processing (Table 2). These phenomena lead to a significant increase in the hardness regardless of the nature of the phases within the polluted material.

As a conclusion, different phases within Ti6Al4V may exhibit overlapping hardness values. The use of overall hardness based on rules of mixtures to validate phase amount may be inadequate, in particular if the hardness values are modified by the physicochemical and metallurgical effects mentioned above [33]. Therefore, Vickers hardness alone is not relevant for microstructure characterization of Ti6Al4V.

### 2.5. A Brief Review on Kinetic Models and Their Limitations with Regard to AM

Several kinetic models applied to solid-state transformations during AM processes can be found in the literature. For diffusion transformations, the Johnson–Mehl–Avrami–Kolmogorov (JMAK) equation determines the amount of α lamellae within α/β_W_ or α/β_C_ structures obtained from β transformation, including incomplete reactions [43,44,45,46,49,69]. This approach has been successfully extended to the prediction of mechanical properties through the size of α lamellae [16,18,24,50].

For the martensitic transformation, the empirical Koistinen–Marburger (KM) equation is often used to predict the amount of α′ resulting from very quick cooling down from the β field [4,43,48,56,69]. However, the setting of the parameters for this model is strongly dependent on the transition points M_s,α′_ and M_f,α′_, or on the critical cooling rates considered for the activation of this transformation. As already mentioned, there is no consensus on these data. In addition, Figure 2 clearly suggests threshold functions depending on $\dot{T}$ contrarily to the threshold points for diffusion transformations that are usually constant and independent of $\dot{T}$. It is probably because of the above assumptions that the recent model developed by Baykasoglu and coworkers [43] cannot predict higher values and abrupt variations within the hardness of a thin-walled DED deposit. Furthermore, several authors decided not to integrate the martensitic transformations in their microstructural model, either because these transformations are poorly understood, or due to the discrepancy within the cooling rates that allow displacive reactions, or because of the challenge of distinguishing and quantifying α′ in the presence of the α phase [49,50]. Moreover, even the very recent study by Liu and Shin [33] presents several limitations concerning the kinetic model. Firstly, the model assumes only a diffusive mechanism for the reverse transformation α → β during heating, and the related critical points are set as fixed values. Then only the CCT approach is considered for β → α/α′ transformation without taking into account the possible isothermal reactions that involve either β or α′ as parent phases. To the authors’ knowledge, no model integrates the case of the RDsT for the heating transformation from α′ phase, in the same way that no model combines both CCT and TTT approaches for solid-phase reactions during cooling.

Gil and Mur [59] established a model for the decomposition of martensite upon annealing, i.e., heating up to a temperature below β_transus_ followed by a holding time. For this model, the parameters were based on the evolution of hardness with annealing time, between a quenched martensitic and subsequent annealed states. Assuming the martensite decomposition to be similar to recrystallization, the precipitation fraction of β by-product is obtained using an Avrami-like equation.

However, the hardness of 330 HV taken as a reference for the quenched conditions seems low compared to that of the fresh martensite. In addition, for the annealed states, hardness values were not measured at the tempering temperature, but after cooling down to room temperature. As a result, an increasing trend up to 410 HV for the hardness was found with increasing annealing temperatures, thus suggesting a possible α-case [70]. Moreover, Gil and Mur [59] mentioned a critical M_f,__α′_ of 800 °C as a ceiling value for the tempering, which is rather well known as the starting point of martensitic transformation [1,38,47,57] during cooling. Therefore, this kinetic model for martensite decomposition [59] based on hardness measurements does not seem reliable.

Phase field models are used to resolve microstructural features in small length scales [71,72,73]. For this purpose, an order parameter based on free energy that represents the state of the entire microstructure is calculated, assuming all the variables to be continuous across the interface [72]. Phase field-base models often tackle both precipitation and dissolution of second-phase particles on the one hand, and diffusive transformations on the other. Therefore, martensitic transformations are ignored [73].

The work from Shi et al. [71] based on multiphysics and multiscale modeling of Ti6Al4V in AM addressed the effect of phase transformations under different cooling rates, via numerical evaluation of elastic properties at the micro- and mesoscopic scales. Although such an approach is useful to determine the residual stresses within AM parts, the martensitic transformation was not considered in this study.

In summary, different phases can be observed in the final microstructure of as-built Ti6Al4V AM parts, as a result of the complex thermal history achieved during manufacturing. However, only a few attempts have been made to use or combine existing kinetic models to predict and validate, the presence of α, α′ and possible β_ret_ at the same time. The main challenge appears to be how to simultaneously take into account the specific mechanisms governing all the phase transformations, and to simplify the thermal history to offer an efficient model coupled with FE simulations. The present paper introduces a novel concept, namely the time-phase transformation-block (hereinafter referred as TTB), which will help numerical teams to select only parts of the thermal history and still keep the key thermal features governing the final microstructure (see Section 3.4).

## 3. Materials and Methods

### 3.1. Material Origin and Processing

**Figure 5:**
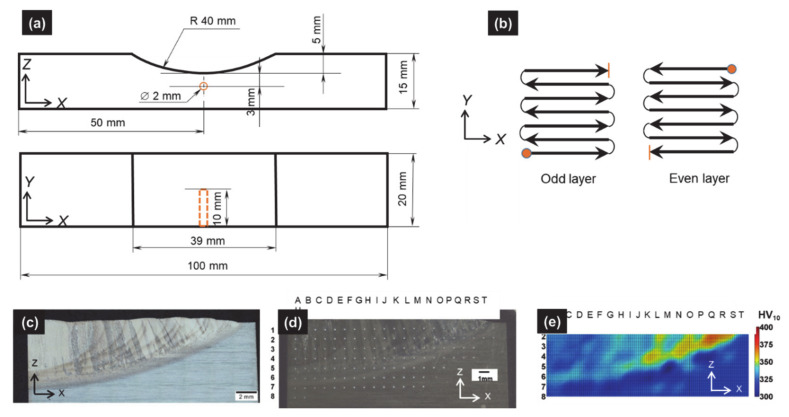
(**a**) Sample geometry and type-k thermocouple location within the substrate; (**b**) path of laser beam for the DTL deposition strategy (7 tracks/layer, and 10 layers); (**c**) view of half deposit in as-built conditions with columnar macrostructure and enhancement of HAZ (dark curved areas) and large nugget in the center (light area); (**d**) indentations grid within the cladded deposit (HV 10); (**e**) Vickers hardness map.

A 5-axis laser cladding system (IREPA LASER, Illkirch, France) equipped with a Nd-YAG laser source of maximum power 2000 W from Sirris Research Centre (SIRRIS, Seraing, Belgium) was used for the sample production. The laser spot has a top-hat energy distribution with a diameter of 1400 µm. The laser power was set at 1100 W, the scan speed at 400 mm/min, and the powder feed rate at 28 mg/s. A type-K thermocouple was inserted 3 mm below the base of the notch at a position corresponding to the mid-length and mid-width (Figure 5a), in order to record the temperature as a function of time. As described in [38], the recorded thermal history is used as a reference to validate the thermal model presented in Section 3.3. More details on the feedstock materials and fabrication process can be found in [5,38].

### 3.2. Experimental Methods

Samples for metallographic observations were cut using a 5-axis wire-electro discharge machining (CHARMILLES Robofil 310, Satigny, Switzerland). The samples were hot-mounted in a resin (STRUERS Citopress, Willich, Germany) and mirror polished down to 1 µm (STRUERS Tegramin, Willich, Germany). Samples were etched using Kroll’s reagent in order to reveal the details of the microstructure. Observations were carried out using both optical microscopy (OM, OLYMPUS BX60M, Olympus Europa, Hamburg, Germany) equipped with a digital camera OLYMPUS UC30 (Olympus Europa, Hamburg, Germany) and a motorized stage) and scanning electron microscopy (SEM, PHILIPS XL30 FEG-ESEM, FEI Company, Hillsboro, OR, USA). Vickers hardness measurements (HV 10) were performed following a grid (Figure 5d), by means of universal hardness (EMCO MC10 010, EMCO-TEST, Kuchl, Austria) device equipped with an electronic cell force. In order to avoid interactions between adjacent indentations, a distance of 1 mm was set between test points. To draw a map of iso-hardness contours (Figure 5e), fictive intermediate points between actual hardness measurements were generated using a “triangle-based cubic interpolation” in MATLAB^®^ software (Mathworks, Natick, MA, USA). Additional details on experimental characterization procedures may be found in [5].

### 3.3. Microstructure Characterization Focusing on Three Points of Interest (POIs)

**Figure 6:**
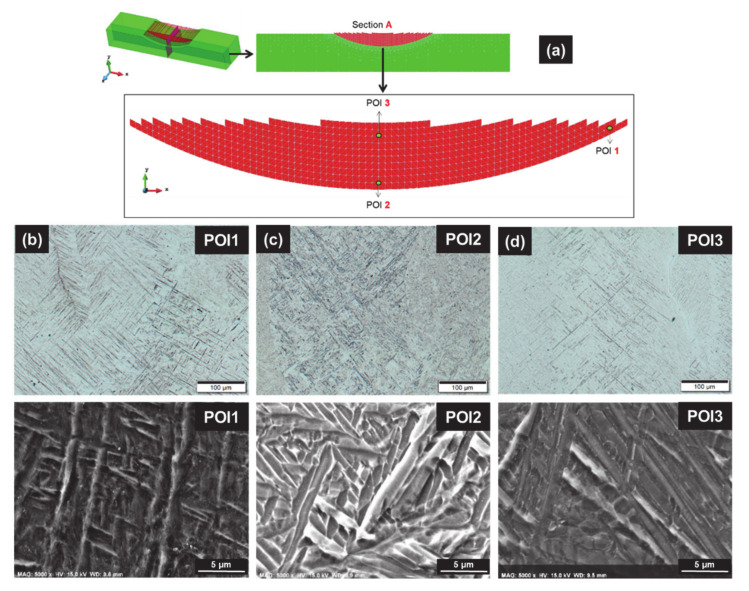
Microstructure in the as-built conditions; (**a**) location of the three points of interest POIs), and FE mesh; (**b**–**d**) light microscope (top) and zoom under SEM (bottom) for POIi—(**b**) POI1: α′ martensite matrix made of thin orthogonal laths, with α_m_ at prior β grain boundary; (**c**) POI2: α/β_W_ basket-weave structure, with few coarsened discontinuous laths of “decomposed” α′; (**d**) POI3: α′ with some neighboring α/β_W_ structure, and α_m_ at prior β grain boundary.

The microstructures of three points of interest, POI1, POI2 and POI3, corresponding respectively to the maximum, the medium and the minimum of Vickers hardness, are given in Figure 6:

- For POI1 (Figure 6b), the matrix is made of orthogonal thin laths of martensite with acicular morphology. α massive (α_m_) is also present at prior β columnar grain boundaries.
- The microstructure in POI2 (Figure 6c) presents fewer thick α′ laths, and typical α lamella not aligned with α′ orthogonal laths. α represents the main phase corresponding to the Widmanstätten structure with a basket-weave morphology (α/β_W_).
- POI3 exhibits both α′ and α_m,_ similar to POI1, with very few α/β_W_ between α′ laths (Figure 6d).

Compared with the constant track length case analyzed in [5], the decreasing track length (DTL) strategy enhances the clad heterogeneity and results in a graded microstructure [2,5,35,36,48] as proved by Vickers hardness (Figure 5e), OM and SEM observations (Figure 6).

### 3.4. Thermal Modeling

**Figure 7:**
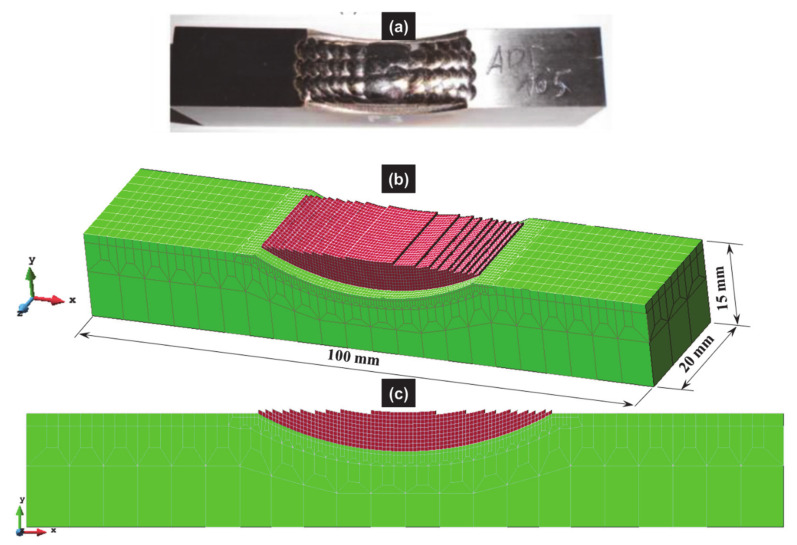
(**a**) Deposit in the as-built conditions, and 3D finite element mesh used for the numerical simulation of the laser cladding process; (**b**) 3D view; (**c**) front view.

The updated Lagrangian FE software called Lagamine developed by the University of Liège to model forming processes [74] was applied here. An 8-node 3D thermo-mechanical element with a reduced integration scheme and an hourglass control technique [75] was used; however only the thermal degree of freedom was activated. The 3D mesh was refined in the deposit and at the top of the substrate, while a coarser mesh was chosen at the bottom of the substrate (see Figure 7).

The material addition was simulated by the element birth technique. Within the model, convection and radiation are considered with the environment where the ambient temperature is 298.15 K. Due to natural convection (free convection of this process), constant heat transfer coefficient (h) was used (h = 52 W/m^2^K). The heat loss due to radiation is described by a single value of emissivity which has been common practice in laser cladding modelling [38]. Here, the emissivity used is ε = 0.8. Here, the DTL strategy to fill the notch was applied while previous study [38] was focused on a constant track length strategy. The distance between the bottom of the groove and the type-K thermocouple location within the substrate was 3 mm here instead of 2 mm in the previous works [38].

The effects of the latent heat of fusion and vaporization was integrated in the definition of an apparent heat capacity and the fluid motion (i.e., Marangoni flow) was not considered, in order to reduce the complexity of the problem.

**Figure 8:**
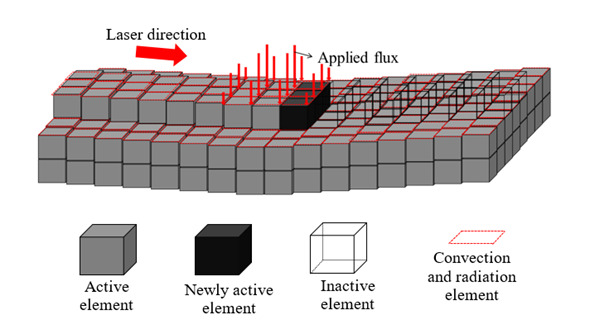
Scheme of element birth and death technique along the curved surface, updated state when the laser has moved (Reprinted with permission from ref. [38]. Copyright 2017 Elsevier).

The initial constant temperature field was defined based on the preheating conditions. The convection and radiation phenomena were considered with the ambient temperature 298.15 K. An element mesh size of 0.5 mm was selected for the deposit, which means a total of nine elements describing the heat flux *q_laser_* loading under the laser beam (see Figure 8):

(1) $$q_{laser}=\beta_{abs},I\;\left(x,\;y,\;z,\;U,\;t\right)$$

where *β*_abs_ is the absorption factor, *I* the laser heat flux density distribution and U the experimental velocity of the laser in the *x*, *y*, *z* directions, respectively. By inverse modeling, a laser absorptivity coefficient of 0.35 was numerically identified based on the experimental temperature curve of the first layers while both convection and radiation coefficients were calibrated with the next layers. The *β*_abs_ value is close to the values given in the literature, ranging from 0.30 to 0.40 [76]. The laser beam velocity and the idle time between tracks and layers were obtained from the experimental conditions.

**Figure 9:**
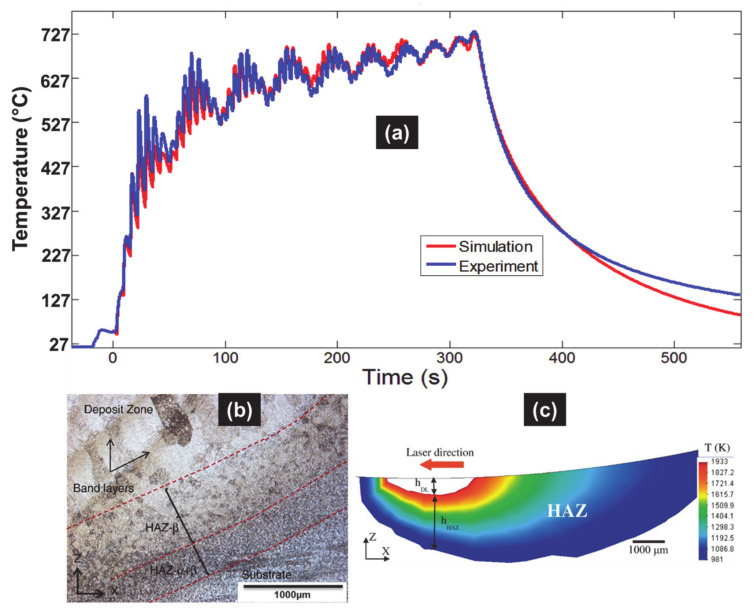
(**a**) Predicted and measured temperature–time curves at the thermocouple inside the substrate for 10 layers. (**b**) Estimation of deposition features (dilution zone, HAZ) at the first layer and the mid-width of the substrate beneath the thick Ti6Al4V deposit obtained by DED, with parameters measured by metallographic inspection (Reprinted with permission from ref. [5]. Copyright 2015 Elsevier); (**c**) parameters predicted using the validated thermal model (Reprinted with permission from ref. [38]. Copyright 2017 Elsevier).

The predicted thermal field was validated by the comparison of the measured and predicted thermal histories (Figure 9) as well as the sizes of the dilution zone and the HAZ within the substrate measured under metallographic inspection. Both checks were important to identify a single set of input parameters.

A difference of around 10% was found. Such a discrepancy can be due to the variable shift within the transformation points of Ti6Al4V alloy with high heating rates under AM processing, while the current simulations use a constant temperature value.

## 4. Results

Based on the validated 3D FE thermal model, the simulated thermal histories for the three POIs were obtained and analyzed hereafter.

### 4.1. Simulated Thermal History on POI1

**Figure 10:**
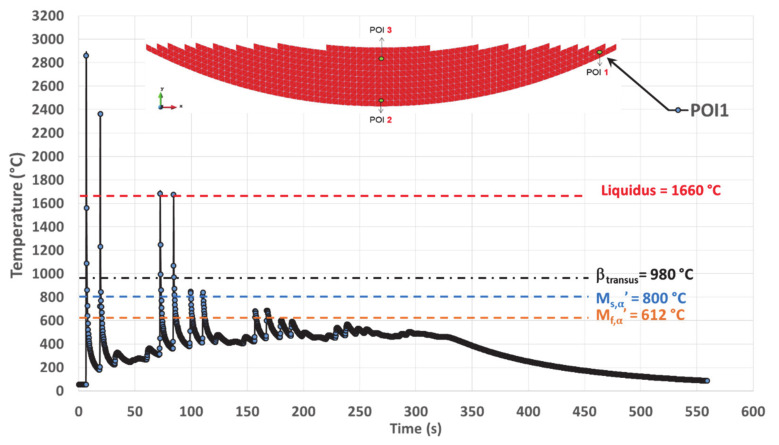
Simulated thermal history of POI1, starting from the material deposition, up to the final cooling at the end of deposition.

This point is located on the edge of the cup and corresponds to the beginning of the deposition (first track). In the thermal history (Figure 10), the first peak, also the highest one, sets the element activation in the simulation. The peaks above the liquidus are paired off. Each pair corresponds to the two adjacent tracks belonging to the same layer, located close to the related POI. The closer the laser beam is to the POI, the higher the peak temperature. If there is a marked difference between two paired peaks, the one with the highest temperature will be that for which the laser beam was closest.

Complete melting is assumed for each peak higher than the liquidus, prior to the rapid cooling to a temperature much lower than β_transus_, thus allowing the solidification to locally occur. New remelting is achieved again if local temperature increases above liquidus. The maximum amplitude of the complete remelting peaks decreases with time (Figure 10 and Table 3).

For POI1, melting is achieved four times before solidifying for the last time, starting from a peak temperature of 1675 °C (Figure 10 and Table 3). The last solidification is achieved with an average cooling rate of 93 °C/s, determined between the maximum and the minimum peaks (Table 3). The method for calculating these cooling rates is explained in Section 5.2.2.

After the last solidification, POI1 experiences a thermal cycling with peak temperatures ranging well below β_transus_ until the deposition stops. The amplitude of the thermal cycling quickly decreases over time because of the building strategy. Indeed, the heat source becomes more and more remote as deposition proceeds, moving from the edge of the cup where POI1 is located to the center of the cup. Simultaneously, the minimum temperature slightly increases with time, up to a plateau around 450 °C as the result of a moderate heat accumulation.

At the end of the deposition process, when the laser beam is turned off, there is a continuous cooling down to the room temperature that follows a typical exponential decay.

### 4.2. Simulated Thermal History on POI2

**Figure 11:**
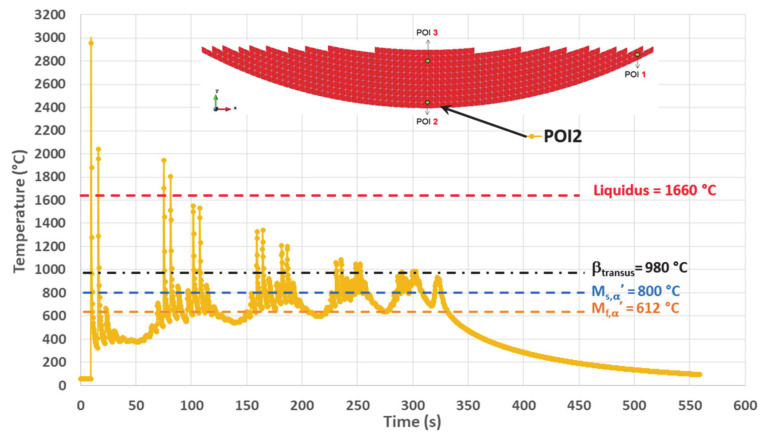
Simulated thermal history of POI2, starting from the material deposition up to the final cooling.

This point, located on the bottom center of the cup, is filled out after POI1 within the same first layer and is re-melted three times (corresponding to the first two layers). The last solidification is achieved starting from a temperature of 1804 °C, under an average cooling rate of 228 °C/s (Figure 11 and Table 3).

Akin to POI1, the remelting peaks within POI2 follow a decreasing trend with time, their values being well above the liquidus. However, the thermal cycles following the last solidification of POI2 present a series of paired peaks whose temperatures are significantly higher than β_transus_, contrarily to what occurs within POI1. These peaks are relative to layers 3 to 7. The last three pairs of peaks (layers 8 to 10) have a maximum either close to β_transus_, or slightly below. Similarly to POI1, turning off the laser beam at the end of deposition process leads to a final continuous cooling down to room temperature.

Thermal cycles of lower amplitude are present between paired peaks characterized by values higher than β_transus_. The maximum of these intermediate peaks remains below β_transus_ and their minimum sometimes falls below M_f,α′_; however, their cooling rate $\left|\dot{T}\right|$ is always lower than the critical cooling rate (CCR) of 20 °C/s (Table 4).

There are more temperature fluctuations within the POI2 thermal history than that of POI1. While the minimum temperature is still increasing gradually up to a plateau, the average temperature exhibits an even more pronounced increasing trend, a phenomenon probably due to the heat accumulation as the result of the building strategy.

### 4.3. Simulated Thermal History on POI3

**Figure 12:**
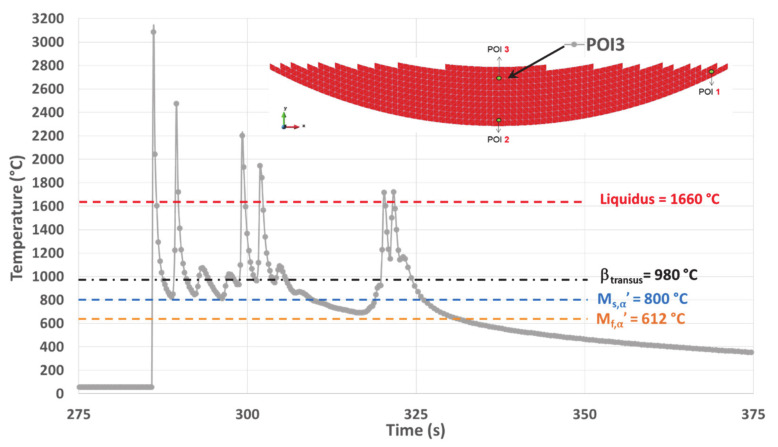
Simulated thermal history of POI3, starting from the material deposition up to the final cooling.

POI3 is re-melted five times as the maximum of the temperature peak passes over the liquidus six times upon heating. This is usually achieved under high heating rates (Figure 12 and Table 3).

The maximum temperature of 3083 °C for the molten liquid is reached when melting the powder for the first time. Nevertheless, this temperature remains below the evaporating temperature of Ti6Al4V (3287 °C, [38]), thus avoiding changes within the composition of the alloy. The corresponding $\dot{T}$ is also at its highest value (12,316 °C/s). Therefore, both the peak temperature and the heating rate achieved for the first melting within POI3, represent the highest values among all the equivalent data obtained within the three POIs. This fact is related to the effect of heat accumulation, more pronounced in POI3 due to the building strategy. A similar result was established in a previous work ([38]) for a point of interest also located in the top center of the cup, but with a constant track length building strategy.

The last melting peak in POI3 has its maximum at 1722 °C. The consecutive $\left|\dot{T}\right|$ upon cooling reaches a value of 612 °C/s, leading to a relative minimum of 1145 °C at the end of the cooling stage. This minimum temperature is well above all critical transition points for solid-phase transformations, which means that the parent β phase should remain untransformed. Then there is an almost instantaneous increase in temperature up to 1170 °C, followed by a slow cooling stage as already observed on both POI1 and POI2.

## 5. Discussion

### 5.1. Introduction of the TTB Concept

As demonstrated in the literature survey of Section 2, predicting an accurate microstructural evolution during AM remains a major challenge. The authors propose the new concept of time-phase transformation–block (TTB) to tackle this issue. More specifically, the TTB approach consists of simplifying the complex thermal histories of the AM process as obtained from a validated thermal model simulation, by cutting them into successive blocks of time. Each block represents a thermal sequence that can be distinguished according to the single-phase transformation mechanism that occurs. The by-products of the previous block of the TTBs are used as new parent phases upon the transition from one TTB to the following one.

For the definition of each TTB, one must focus on a specific thermal sequence. Upon heating, the TTB should highlight CHT, while CCT should be considered during cooling stages. Under quasi-isothermal conditions, TTT are considered together with the mechanism related to phase transformation kinetics. In the latter case, it is possible to start from a higher temperature to cool down to a lower and fixed temperature, or to heat up to a plateau and hold for a definite time. Hereafter, the implementation of the TTB concept on the case presented in Section 3 is described.

### 5.2. Application of the TTB Concept to the Microstructure Evolution within POIs

#### 5.2.1. Setting of TTBs

The TTB concept is intended to cut the complex thermal histories observed in AM into simple segments. This sectioning eases the transposition of existing predictive models, with some improvement as they do not currently take into account most of the phenomena involved either by high thermal gradients or short dwell times. To guide numerical models without excessive simplification, the new concept TTB is developed hereafter.

The threshold values defined in Section 2 (β′_transus_, α′_transus_, β_s_, β_f_, M_s,αm_, M_s,α′_, M_f,α′_) for phase transformations have to be determined and reported for each POI case (Section 5.2.2). Upon heating, a temperature range for β′_transus_ above β_transus_ is considered for defining the end of the RDfT yielding β phase regardless of the starting α or α′ phase. Although the onset of the RDfT (α′_transus_) increases with $\dot{T}$, it will not be taken into account here. Indeed, hereafter, the focus is only on specific cases for which the transformations are complete, thus assuming a peak temperature higher than β′_transus_. In addition, displacive limits β_s_ and β_f_ well above β′_transus_ are considered for RDsT of α′.

For DDsT occurring upon cooling, M_s,αm_ and M_s,α′_ are set at 893 and 800 °C, respectively, the latter value being chosen in view of its good agreement with the thermodynamic approach and because it is composition-dependent [8,47,57]. M_f,α′_ is set at 612 °C as the average of the four empirical values previously mentioned in Section 2.2.

#### 5.2.2. Application of TTB Concept to Simulated Thermal Histories

The new TTB concept is applied on simulated thermal histories of Section 3 to highlight metallurgical phenomena.

It is worth nothing that heating rates $\dot{T}$ of the first peaks calculated by the FE simulation do not have a real physical meaning (Peak 1 in Table 3). In fact, the element birth technique used during simulation activates a new element only when it is heated by the laser beam and does not accurately model all the phenomena related to powder grains.

**Figure 13:**
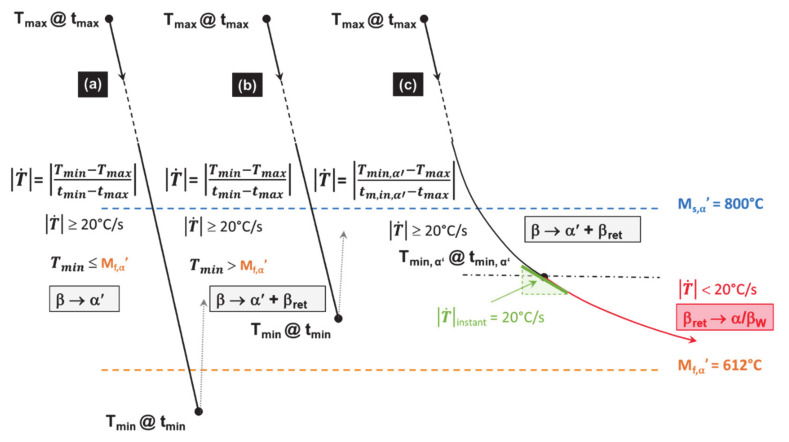
Methods determining the average cooling rates $\left|\dot{T}\right|$ in TTB1 and TTB3. Case (**a**) allows a complete martensite transformation; (**b**) a partial one; and (**c**) a combined transformation starting with a displacive mechanism before switching to a diffusive mode, when $\left|\dot{T}\right|$ is lower than the CCR of 20 °C/s.

The methods for calculating the average cooling rates $\left|\dot{T}\right|$ used within both TTB1 and TTB3 are explained in Figure 13. In this case, $\left|\dot{T}\right|$ are computed between M_s,α′_, M_f,α_’ or from M_s,α′_ to the minimum of temperature reached (Table 3).

Table 3 and Table 4 summarize the parameters characterizing each TTBs for the three POIs. They are key input data for the application of the TTB methodology. These values help in enhancing the transformation mechanism, which can change from displacive to diffusive when $\left|\dot{T}\right|$ rapidly decreases during cooling. In this case, the switching time is set when the instant $\left|\dot{T}\right|$ becomes lower than the CCR of 20 °C/s.

**Figure 14:**
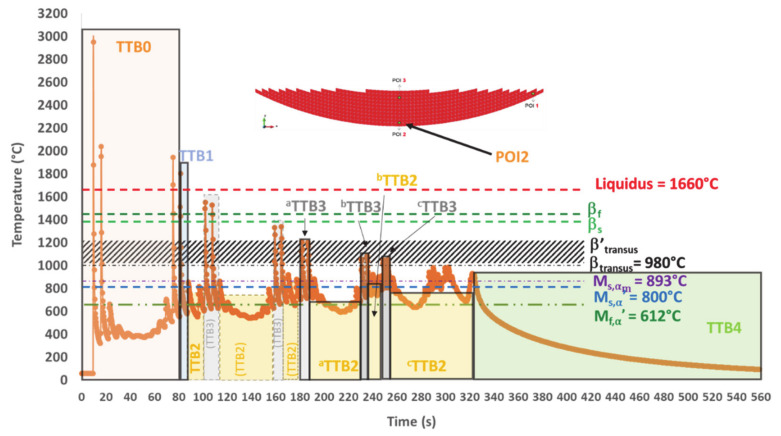
TTB sequence within the simulated thermal history in POI2; dashed line boxes for phase transformations wiped out during processing, and solid line boxes for phase transformations products remaining in the final state (three relevant TTB3s and three relevant TTB2s are underlined).

**Figure 15:**
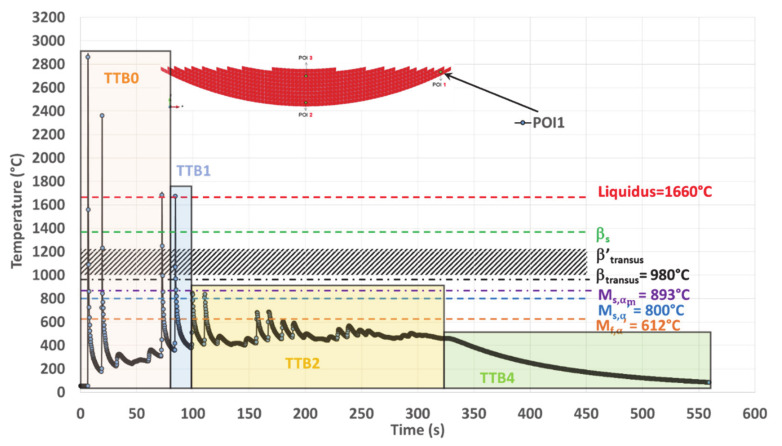
TTB sequence within the simulated thermal history on POI1 where TTB3 is missing due to a peak temperature well below transition points after TTB1 and TTB2.

**Figure 16:**
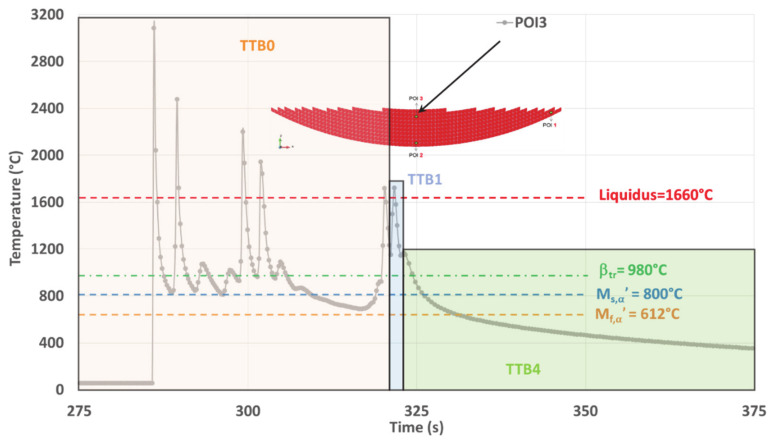
TTBs sequence within the simulated thermal history on POI3 where both TTB2 and TTB3 are missing due to quasi-continuous cooling straight from the last remelting, down to room temperature.

The first focus is on POI2 (Figure 14), which exhibits the most complex thermal history before transposition to other POIs (Figure 15 and Figure 16). Five distinct types of TTBs are defined as follows:

- TTB0 corresponds to the initial stage comprising a series of superheating peaks with their maximum above the liquidus, and during which the material previously solidified, is systematically remelted.
- TTB1 identifies a single peak corresponding to the last solidification event. Its maximum temperature *T_max_* is higher than the liquidus. The *T_min_* value reached after the peak and the cooling rate ${\dot{T}}_{mean}$ between these extrema determine the new parent phase “1” (α′, α_m_, α/β_W_ or _βret_).
- TTB2 covers the time periods where a succession of peaks have their maximum lower than β_transus_. The average of this saw-tooth thermal profile provides a representative isothermal value.
- TTB3 corresponds to the paired peaks with both steep temperature increases and decreases. Their maxima, well above β_transus_, generate partial or complete reverse transformation into β, depending on the highest temperature achieved during heating. The minimum temperature and the cooling rate achieved during the cooling stage of the last peak set the provisional microstructure at the end of TTB3.
- TTB4 is the last continuous cooling down to room temperature, at the end of the AM process. Solid state transformations can still occur during this period, if the thermodynamic conditions allow it.

##### POI2 Analysis

All the TTBs applied to POI2 are illustrated in Figure 14.

Within TTB0, POI2 is consecutively melted three times. The third melting peak occurs about 75 s after the element activation. The first peak is the highest, probably because it is the one for which the laser beam is just right in the POI during the deposition of the first layer. It can be noted that both $\dot{T}$ and $\left|\dot{T}\right|$ related to those remelting peaks are very high (Table 3). The cooling stage during the first peak occurs under a $\left|\dot{T}\right|$ of 488 °C/s down to a temperature that is well below M_f,α′_. Martensite is necessarily formed after this peak, but it disappears by means of a diffusionless transformation into β, a phase which itself is almost immediately re-melted when the laser beam approaches POI2 again, for the deposition of an adjacent track within the same first layer. In the same way, martensite is again directly formed during the cooling associated with the second melting, as a result of a displacive transformation of the parent β formed earlier during solidification. Such a statement is in good agreement with [52], where mechanisms for quick heating transformations up to remelting have been evidenced by SXRD. This second martensite will in turn undergo a thermal cycling that can lead to further modifications. However, they are neglected here, as another complete remelting will be achieved later on. Indeed, the third and fourth peaks correspond to the laser beam passing over POI2 and melting this material point again, when building two adjacent tracks belonging to the second layer. In addition, it can also be observed in Table 3 that $\dot{T}$ does not necessarily follow a monotonic evolution.

Solid-phase transformations occurring within TTB0 are not considered because the complete remelting achieved later wipes out the solid phases. However, such a thermal cycling up to complete remelting may be interesting for studies related to melt pool geometries, and primary β grain size and morphology, including epitaxial growth.

After TTB0, a single TTB1 follows. It identifies the single peak corresponding to the last remelting of POI2 (see its characteristics in Table 4). It may be noted that a $\left|\dot{T}\right|$ of 228 °C/s is achieved during cooling, down to a minimum temperature of 579 °C, which is lower than M_f,α′_. Therefore, the martensitic transformation is complete at the end of TTB1.

Following TTB1, there are six TTB2s intercalated by five TTB3s. The first three TTB2s are followed by TTB3s, where a complete transformation of the existing α′ phase into β without recrystallization is assumed (see Appendix A for details). This hypothesis seems reliable, as the peak temperatures within these TTB3s are higher than β′_transus_. One can thus forget these three first TTB2s (see Figure 14 and Table 4). In addition, the temperature peaks corresponding to the tracks closest to POI2 for the last three layers (8 to 10) have their maximum lower than β_transus_. As previously established (Section 2.1), the transition point such as β′_transus_ is higher than equilibrium reference point β_transus_ due to the $\dot{T}$ effect. In addition, the minimum residence time for an isothermal transformation is not achieved under a single peak (see Appendix A for details).

One can identify the first relevant TTB3, noted as ^a^TTB3, whose product phases will remain afterwards. In particular, during the second cooling, the minimum temperature reached is lower than M_s,α′_ and still higher than M_f,α′_, thus leading to an incomplete martensitic transformation (Table 4, ^a^TTB3). Both α′ and β_ret_ are then submitted to an isothermal annealing (^a^TTB2). α′ undergoes a decomposition while β_ret_ leads to α/β_W_ by a diffusional transformation (see TTT diagram [4,15,44,45]). During ^b^TTB3, an incomplete reverse transformation of parent α and α′ phases into β happens because the maximum peak is lower than β′_transus_. Then, the newly formed β undergoes a martensitic transformation upon cooling. The progression of this martensitic transformation is less marked than for ^a^TTB3 since the minimum temperature achieved (746 °C) is higher than the previous one (718 °C) (Figure 14 and Table 4). The phases present at the end of ^b^TTB3 then undergo an isothermal sequence ^b^TTB2. This cyclic approach alternating TTB3 and TTB2 decreases the amount of martensite since its decomposition takes place within ^a,b,c^ TTB2s, while new α′ is expected only in ^a,b^ TTB3s. Indeed, T_min_ for ^c^ TTB3 is higher than M_s_. In the meantime, a complete transformation of β_ret_ into α/β_W_ during ^c^TTB2 is achieved at 777 °C (Table 4). During the final cooling (TTB4), no transformation occurs because there is no β phase left, and there is no phase decomposition during cooling. POI2 exhibits the lowest hardness among the three POIs due to the relatively high amount of α/β_W_ that is obtained under quasi-isothermal conditions (Figure 5e and Table 4).

In conclusion, the thermal history of POI2 with peak temperatures higher than β_transus_ and subsequent high-temperature isothermal annealing enhances the conditions for recrystallization.

##### POI1 Analysis

POI1 is melted four times, and the maximum temperature of the melt follows a decreasing trend (Table 3). Among the melting events, the first three ones occur within TTB0, and the last is related to TTB1 (Figure 15).

Both α_m_ and α′ martensite are formed at the end of TTB1 (Table 4) due to an average cooling rate of 93 °C/s, down to 380 °C, which is well below M_f,α′_. During TTB2, martensite is submitted to an annealing at a low average temperature (487 °C) during 228 s, which is too short to allow its decomposition [11,16,22,24,45,59]. POI1 ends its cooling stage with TTB4, during which no phase transformation is expected. The hardness in POI1 is among the highest values achieved within the deposit (Figure 5e and Table 4).

In conclusion, the thermal history of POI1 with peak temperatures lower than β_transus_ and subsequent low temperature isothermal annealing enhances the conditions for martensite decomposition.

##### POI3 Analysis

For POI3, TTB1 does not allow the transformation of β because the minimum temperature levels reached under the high $\left|\dot{T}\right|$ remain higher than the transition points. Phase transformations take place during TTB4 that follows TTB1, as both the isothermal-like TTB2 and the reheating TTB3 are missing (Figure 16).

The final cooling rate (CR) continuously decreases, starting with a value higher than the CCR of 20 °C/s that allows martensitic transformation. However, CR falls below this CCR value, stopping the martensitic transformation before its completion, i.e., for T > M_f,α′_. As a result, the final microstructure within POI3 originates from two distinct successive mechanisms. First, a DDsT that allows the parent β phase to transform into α′. This martensitic transformation continues as long as $\left|\dot{T}\right|$ remains higher than 20 °C/s. Second, a DDfT that allows βret to transform into α/β_W_. This two-stage phase transformation is in good agreement with the mixed (or dual) matrix observed within POI3 (Figure 6d), where a hardness between that of POI1 and POI2 is achieved (Figure 5e and Table 4).

The following continuous cooling stage down to room temperature occurring within POI3 differs from thermal cycling made of several successive heating and cooling sequences observed on both POI1 and POI2. Therefore, the microstructure associated with POI3 is likely to lead to reduced dislocation densities, which explains why a relatively low hardness is achieved within this point.

In conclusion, the thermal history of POI3 with complex CCT achieved due to the suppression of subsequent reheating and isothermal annealing enhances the conditions for change within the transformation mechanism.

### 5.3. Flowchart Describing the Evolution of Microstricture during DED Process Based on TTBs

In summary, the thermal history obtained by FE simulation is analyzed through TTB concept. It selects the relevant thermal features required to identify the solid-phase transformations occurring during the process. An accurate prediction of the microstructural evolution at any point of a multilayered Ti6Al4V thick deposit manufactured by DED should be developed based on this concept.

**Figure 17:**
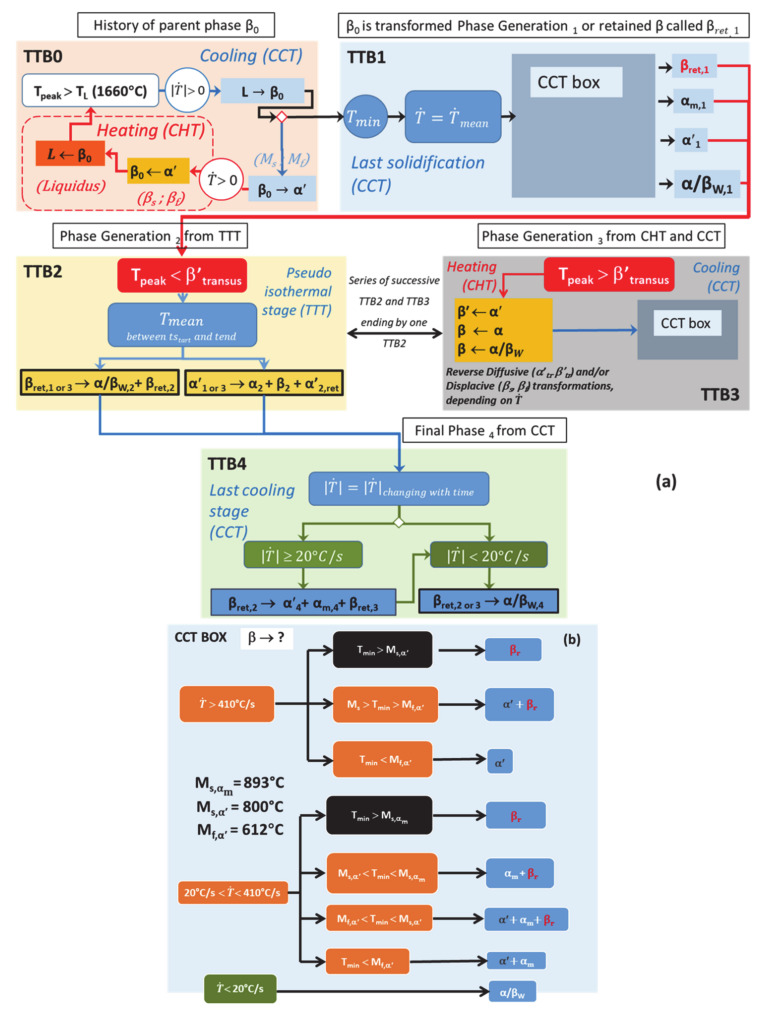
(**a**) General time-transformation-blocks (TTBs) based on thermal histories with related phase transformations occurring during DED; (**b**) highlighting of CCT Box transformations occurring within TTB1 and TTB3.

For each block of the TTB concept, the type of transformation according to their critical temperatures and critical thermal rates ($\left|\dot{T}\right|$ or $\dot{T}$) is summarized in the flowchart of Figure 17.

Key data for displacive transformations occurring under continuous cooling conditions have been set, such as critical points (M_s,αm_, M_s,α′,_ M_f,α′_) and related critical cooling rates $\left|\dot{T}\right|$. The most relevant values for the transition points, or the range of values for $\left|\dot{T}\right|$, have also been identified. These values (key input for any transformation model) have been achieved thanks to a critical review of the literature followed by an experimental validation on a manufactured part.

For the diffusive transformations, critical cooling rates remain to be determined. The influence of the heating rate $\dot{T}$ on both the mechanism for reverse transformations $\alpha /\alpha^{\prime }\to \beta$ and the critical transition points are also highlighted. These parameters still require additional experimental works. A function of the heating rate for the critical transition temperature of RDfT, as well as constant values for RDsT when $\dot{T}$ reaches a threshold value, are still to be identified.

However, the flowchart of Figure 17 allows numerical teams to easily implement a set of phenomenological equations based on physical roots. Therefore, improvement within the so-called mechanistic models can be achieved thanks to the specific highlighting of the mechanisms that govern each type of transformation in solid phase [73].

From a different perspective, machine learning (ML) has been used in all steps of AM as a tool that allows for rapidly predicting the microstructure, properties and defects without dealing with the solution of complex equations based on phenomenological understanding [73]. A combination of the TTB concept with rapidly evolving ML approaches opens up very promising prospects for future works. Indeed, such synergy can be beneficial at three different levels. Firstly, ML has established itself as a robust tool for quantitative phase analysis [77,78,79], thus facilitating the collection of the large amounts of data needed for the careful validation of enhanced thermo-metallurgical FE models. Second, Bayesian approaches combined with ML have already been proved powerful for parameters identification, and this could be applied to the set of presently unknown material parameters within the TTB concept [79]. In addition, it has recently been established that a physics-informed feature engineering enables ML with limited data, thus allowing us to model the effects of metallurgical processing variations on the temperature range of a martensitic transformation [80]. Ultimately, ML can be used in its own right to predict phase transformations either by diffusion [81] or under a displacive mode [82,83], while overcoming current limitations related to the computational cost of FE models. Yet, to reach this goal, properly validated FE models are needed to generate the large amount of data required to train the ML approaches and to reduce the heavy characterizations and experimental campaigns.

With these prospects in mind, the TTB approach provides an easy way to understand the transformation mechanisms and facilitates an efficient implementation of the phenomenological models such as JMAK and KM with a single data set adapted to the AM of Ti6Al4V. Indeed, in the literature, one difficulty of these models is the identification of a single set of material parameters as the parent phases have different compositions. Here the TTB approach and the good understanding of the transformation mechanisms will help to set functions for the input parameters which will enhance the accuracy of the predictions.

## 6. Conclusions

The following conclusions can be drawn:

- A thick multilayer deposit was fabricated by DED using a decreased track length strategy, which allows us to obtain a heterogeneous microstructure. This case can be representative of a repair method.
- The use of a validated thermal model provides simulated thermal histories at different POIs, each corresponding to a specific microstructure within the DED deposit.
- The critical analysis of thermal histories using the new TTB concept highlights the mechanisms that drive phase transformations, either during solidification and possible remelting sequences, or later in the solid state.
- In this article, the identification of the transformation mechanisms allows a qualitative explanation of the presence of any phase within the deposit, using the concept of TTB.
- The TTB concept will help numerical teams to adapt simple models like JMAK and KM to predict the number of phases during AM. The flowchart of Figure 17 identifies critical data and sequences that should be taken into account in these models.

## Figures and Tables

**Table 1:** Literature summarizing correlations between structure hardness of Ti6Al4V, for classical manufacturing processes.

Processes	Thermomechanical Treatment	Macrostructure	Microstructure	Hardness Range (HV)	Comments Related to Mechanical Properties	References
Castings	Slow cooling from the melt	Coarse structure	Coarse α/β_W_ with large α lamellae α_P_ along prior β grain boundaries with α/β_W_ inside grains	320–345	Lower mechanical and fatigue properties Porosities	[62,64,65,66]
Wrought	Extensive mechanical working within (α + β) field + mill-annealed, prior to furnace cooling	Equiaxed structure	Equiaxed α grains with intergranular short-rod β Large α/β_C_	330–370	Hardness scattering related to both the forging temperature and the grain size	[55,65,66]
		Bimodal structure	Granular α_P_ and thin intergranular α/β_W_ lamellae	265–295	Hardness decreases with increasing amount of α_P_ (HV max at 10% of α_P_)	[67]
Solution Treated Quenching (STQ)	Up to 1h above β_transus_ prior to water quenching	Needle-like	Lath and twinned α′ + (β_retained_)	345–420	Hardness increases with decreasing lath size	[55,62,64]
Ageing after STQ	Several hours between 480 °C and 750 °C, prior to furnace cooling			345–380		[61,62]

**Table 2:** Literature summarizing correlations between structure hardness of Ti6Al4V, for AM processes.

Processes	Incident Energies (J/mm)	Macrostructure	Microstructure	Hardness Range (HV)	References
DED	≤60	Columnar structure with epitaxial β grains	Needle-like α′ + (β_retained_)	310–400	[5,13,68]
	(165–480)		α′ + α/β_W_ α/β_W_ + α_GB_	315–370	[5,13,68]
L-PBF	(0.20–1.05)		α′ Fine α′ with acicular laths α/β_W_	320–400 *	[13,19,68]
EB-PBF	≤0.1		Prominent acicular α plates and β α/β_W_ + α_GB_	330–450 *	[13,18,65]

* Oxygen content increases the hardness.

**Table 3:** FE predicted main peak temperatures, heating ($\dot{T}$) and cooling rates ($\left|\dot{T}\right|$) within TTB0 and TTB1 for the three points of interest (POIs).

Points of Interest	Computed Thermal Parameters	Peak 1	Peak 2	Peak 3	Peak 4	Peak 5	Peak 6
POI1	T (°C) $\dot{T}$ (°C/s) $\left|\dot{T}\right|$ (°C/s)	2860 11,390 228	2362 2956 171	1685 1157 120	1675 1396 93	847 * 495 42	-
POI2	T (°C) $\dot{T}$ (°C/s) $\left|\dot{T}\right|$ (°C/s)	2952 11,763 448	2038 2325 299	1944 1457 271	1804 1272 228	1552 * 961 190	1529 * 1225 184
POI3	T (°C) $\dot{T}$ (°C/s) $\left|\dot{T}\right|$ (°C/s)	3089 12,316 848	2477 2235 611	2201 2582 569	1944 1990 457	1719 1619 600	1722 1155 612

* assumed not to be remelted.

**Table 4:** Thermal characteristic and time-based features related to TTB1 to TTB4 as calculated by simulated thermal histories of the three POIs and related local Vickers hardness (VH) values.

Computed Thermal Features and Hardness	POI2	POI1	POI3
T_max_ (°C) @ time (s)	1804 °C @ 82 s	1675 °C @ 84 s	1722 °C @ 322 s
T_min_ (°C) @ time (s)	579 °C @ 87 s	380 °C @ 98 s	1145 °C @ 323 s
Average cooling rate ($\left|\dot{T}\right|$ °C/s) achieved during the last solidification stage	228 °C/s	93 °C/s	612 °C/s
T_mean_ (°C) @ Equivalent holding time (s)	^a^ 669 °C ± 67 @ 39 s ^b^ 829 °C ± 56 @ 09 s ^c^ 777 °C ± 97 @ 66 s	487 °C ± 56 @ 228 s	-
Incubation time (s) for β → α/β_W_ transformation under TTT (t_1%_) @ T_mean_ (°C) [44]	^a^ 1 sec @ 669 °C ^b^ 5 sec @ 829 °C ^c^ 1 sec @ 777 °C	30 sec @ 487 °C	-
Time (s) for progress transformation β → α/β_W_ under TTT (t_50%_) @ T_mean_ (°C) [44]	^a^ 7 sec @ 669 °C ^b^ 10 sec @ 829 °C ^c^ 9 sec @ 777 °C	700 sec @ 487 °C	-
T_max_ (°C) and related$\;\dot{T}$ (°C/s) achieved on the first of the two peaks during heating	^a^ 1203 °C @ 868 °C/s ^b^ 1057 °C @ 660 °C/s ^c^ 1049 °C @ 462 °C/s	-	-
T_min_ (°C) and related $\left|\dot{T}\right|$ (°C/s) achieved on the second of the two peaks during cooling	^a^ 718 °C @ 219 °C/s ^b^ 746 °C @ 173 °C/s ^c^ 804 °C @ 160 °C/s	-	-
Starting peak temperature T_end_ (°C) @ time (s)	930 °C @ 322 s	461 °C @ 326 s	1170 °C @ 323 s
First temperature (°C) at which instant $\left|\dot{T}\right|$ cooling rate falls below 20 °C/s @ time (s)	661 °C @ 330 s	$\left|\dot{T}\right|$ always under 20 °C/s	642 °C @ 331 s
Local VH on POIs (HV 10)	327	370	331

* Indices ^a^, ^b^ and ^c^ are related to the successive relevant TTB2 (4th, 5th, 6th) and TTB3 (3rd, 4th, 5th) occurring on POI2 and during which all or some of the solid phases already formed remain within the subsequent thermal cycles (see Figure 14).

**Figure A1:**
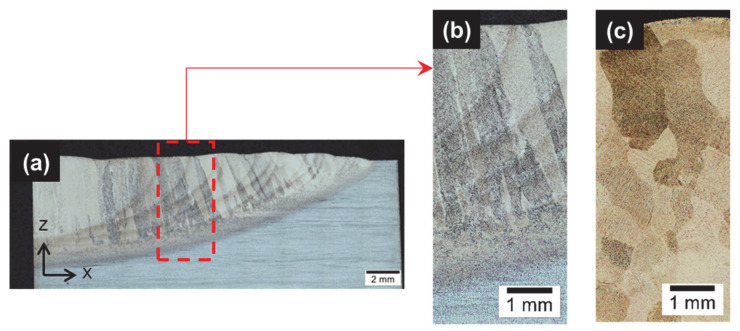
(**a**) Overview of half deposit in as-built conditions with enhancement of HAZ (dark curved areas) and large nugget in the center (light area) (Reprinted with permission from ref. [5]. Copyright 2015 Elsevier); (**b**) close-up view of the inset within (**a**), showing the prior columnar β grains in the as-built deposit; (**c**) β columnar grains turned into equiaxed due to recrystallization after furnace reheating to 1000 °C/30′ and later transformed into α′ due to subsequent water quenching.

## Data Availability

Not applicable.

## Nomenclature

***Phase types***	
*α*	Alpha phase
*α-case*	Oxygen-enriched layer within Alpha phase
*α′*	Martensite
*β*	Beta phase
*α + β*	Complex dual structure made of Alpha and Beta phases
*α/β_C_*	Alpha/Beta with colony morphology
*α/β_W_*	Alpha/Beta Widmanstätten, having a basket-weave morphology
*α_GB_*	Alpha grain boundary formed prior to intragranular *α* or *α/β_W_*
*α_m_*	Alpha massive formed at grain boundaries, prior to intragranular martensite.
*α_P_*	Primary Alpha phase present in the bimodal structure
*β_retained_, β_ret_*	Fraction of untransformed parent *β* phase
***Transition points***	
*Liquidus*	1660 °C, end of the melting for the solid (heating), or onset of solidification for the melt (cooling)
*Solidus*	1590 °C in equilibrium conditions, onset of the melting for the solid (heating) or the end of solidification for the melt (cooling)
*α_transus_, α_tr_ or T_Dissolution_ T_Diss_*	708 °C, onset of α → β or end of β → α reactions under quasi equilibrium conditions. α phase starts to dissolve from this point during heating stage
*α′_transus_, α′_tr_*	Onset of α → β and α′ → β transformation for quick heating rate
*β_transus_ , β_tr_*	980 °C, end of α → β or onset of β → α transformations under quasi equilibrium conditions
*β′_transus_ , β′_tr_*	*>β_tr_* , onset of α → β transformation during quick heating, the same point is also considered for α′ → β.
*M_s,αm_*	893 °C onset of β → α_m_ transformation for cooling rate ranging between 410 and 20 °C/s
*M_f,αm_*	End of β → α_m_ transformation (very close to *M_s,αm_*)
*M_s,α′_*	800 °C onset of β → α′ transformation for rapid cooling >20 °C/s
*M_f,α′_*	612 °C end of β → α′ transformation for rapid cooling >20 °C/s)
*β_s_*	Onset of α′→ β transformation during a steep heating
*β_f_*	End of the displacive α′ → β transformation during steep heating.
***Key temperatures, temperature rates and times***	
$\dot{T}$	Average heating rate between T_min_ and T_max_ on the rising section of the heating curve, for the first peak of TTB3. (°C/s)
$\dot{\left|T\right|}$	Average cooling rate between T_max_ and T_min_ on the decreasing section of the cooling curve, for the single peak of TTB1 or for the second peak of TTB3 (°C/s)
$\dot{\left|T\right|}$_*inst,4*_	Instantaneous cooling rate calculated during final slow down cooling stage within TTB4 (°C/s)
*T_start, 4_*	Starting or maximum peak temperature for the slow down cooling stage related to TTB4 (°C)
*T_crit, 4_*	First temperature at which $\dot{\left|T\right|}$*_inst,4_* falls below the critical cooling rate of 20 °C/s (°C)
*T_max_*	Maximum temperature at the starting point for the fast cooling stage within TTB1 (single peak), or the maximum temperature achieved during the fast heating stage within TTB3 among the two existing peaks (°C)
*T_min_*	Minimum temperature corresponding to the end point for the fast cooling stage within TTB1 (single peak), or minimum temperature achieved during the fast cooling stage within TTB3 for the second of the two existing peaks (°C)
*T_mean_*	Average temperature over the total length of pseudo-isothermal cycle (only for TTB2) (°C)
*T_peak_*	Maximum temperature achieved for any steep heating occurring during TTB0, TTB2 and TTB3 (°C)
*t_0_*	Time at which the deposition within a given POI begins (s)
t_1%_, t_50%_	Respectively the incubation and half the progress reaction times to achieve isothermal transformation β → α/β_W_ within TTB2
t_start_, t_end_	Respectively the start and the end times for average temperature (*T_mean_*) used on the pseudo isothermal plateau within TTB2
Other features	
POI1, 2 or 3	Points of interest 1, 2 or 3
TTB1 to TTB4	Time-phase-Transformation-Block 1, 2, 3 or 4
^a,b,c^TTBi	a, b and c identifies the more relevant peak within TTBi as its related phase transformations remain in the final stage
*α_i_, α_m,i_, α′_i_, α/β_W,i_, β_i_, β_ret,i_*	Index i identifies the related TTBi when the phase is formed

## Abbreviations

The following abbreviations are used in this manuscript:

AM	Additive manufacturing
CCR	Critical cooling rate
CCT	Continuous cooling transformations
CHR	Critical heating rate
CHT	Continuous heating transformations
CTL	Constant track length
DDfT	Direct diffusive transformation
DDsT	Direct displacive transformation
DED	Directed energy deposition
DTL	Decreasing track length
EBDT	Element birth and death technique
EB-PBF	Electron beam powder bed fusion
FE	Finite element
HAZ	Heat-affected zone
IE	Incident energy
IT	Isothermal transformation
JMAK	Johnson–Mehl–Avrami–Kolmogorov
KM	Koistinen–Marburger
LM	Light microscopy
L-PBF	Laser powder bed fusion
ML	Machine learning
RDfT	Reverse diffusive transformation
RDsT	Reverse displacive transformation
SEM	Scanning electron microscopy
STH	Simulated thermal history
TTB	Time-phase transformation-block
TTT	Time–temperature transformations
VH	Vickers Hardness

## Appendix A. Conditions for Recrystallization Occurrence during Reheating

A conventional heat treatment was performed to better understand the mechanism of the reverse phase transformation occurring upon heating when martensite is already present in a part of the deposit during DED. For this purpose, a solution heat treatment was carried out under argon on a small sample extracted from the as-built deposit of Figure A1a. After heating up to 1000 °C for 30 min and water quenching, the original columnar grains (Figure A1b) present in the as built-part become martensitic with equiaxed grains (Figure A1c) smaller in size than the prior β columnar grains.

An average hardness of 377 ± 4 HV10 was measured, which is either due to grain refinement, or to high dislocation densities, twins or stacking faults [21,26,55,60,62]. Recovery usually takes place prior to recrystallization whose rate increases with the dislocations density [68,84]. Both recovery and recrystallization kinetics are thermally activated [23,84]. After a sufficient residence time at high temperature, the quenched sample (Figure A1c) undergoes nucleation and growth of newly formed β grains [35,55]. Additional strengthening/hardening is achieved due to rapid water cooling from β field that suppresses diffusion, leading to supersaturated α′ that contains twins. The nanotwin boundaries are a more effective obstacle to slip than ordinary high-angle boundaries between prior β grains.

However, it must be concluded that the steep thermal cycles with short residence times achieved during DED do not induce any recrystallization within the prior β-grains that grew epitaxially, leading to a coarse columnar macrostructure (Figure A1b) observed in the as-built samples.
